# Two-Stage Water Jet Landing Point Prediction Model for Intelligent Water Shooting Robot

**DOI:** 10.3390/s21082704

**Published:** 2021-04-12

**Authors:** Yunhan Lin, Wenlong Ji, Haowei He, Yaojie Chen

**Affiliations:** 1 College of Computer Science and Technology, Wuhan University of Science and Technology, Wuhan 430000, China; yhlin@wust.edu.cn (Y.L.); wenlong-ji@outlook.com (W.J.); hehaowei@126.com (H.H.); 2 Hubei Province Key Laboratory of Intelligent Information Processing and Real-time Industrial System, Wuhan 430000, China; 3 Institute of Robotics and Intelligent Systems, Wuhan University of Science and Technology, Wuhan 430000, China

**Keywords:** intelligent water shooting robot, pitch angle estimation, online learning, jet trajectory, back-propagation

## Abstract

In this paper, an intelligent water shooting robot system for situations of carrier shake and target movement is designed, which uses a 2 DOF (degree of freedom) robot as an actuator, a photoelectric camera to detect and track the desired target, and a gyroscope to keep the robot’s body stable when it is mounted on the motion carriers. Particularly, for the accurate shooting of the designed system, an online tuning model of the water jet landing point based on the back-propagation algorithm was proposed. The model has two stages. In the first stage, the polyfit function of Matlab is used to fit a model that satisfies the law of jet motion in ideal conditions without interference. In the second stage, the model uses the back-propagation algorithm to update the parameters online according to the visual feedback of the landing point position. The model established by this method can dynamically eliminate the interference of external factors and realize precise on-target shooting. The simulation results show that the model can dynamically adjust the parameters according to the state relationship between the landing point and the desired target, which keeps the predicted pitch angle error within 0.1°. In the test on the actual platform, when the landing point is 0.5 m away from the position of the desired target, the model only needs 0.3 s to adjust the water jet to hit the target. Compared to the state-of-the-art method, GA-BP (genetic algorithm-back-propagation), the proposed method’s predicted pitch angle error is within 0.1 degree with 1/4 model parameters, while costing 1/7 forward propagation time and 1/200 back-propagation calculation time.

## 1. Introduction

Intelligent water shooting robot technology has a wide range of public safety applications [1,2,3] (firefighting, maritime affairs, anti-terrorism, etc.). The traditional water shooting robot [4,5,6] only includes a water jet and a water pump, and the aiming and locking process of the desired target is done manually, resulting in low efficiency and accuracy of the system. In addition, some applications require the water shooting robot to monitor and automatically attack targets in real-time, such as building fire extinguishing systems, which makes traditional water shooting robots unable to perform this task. Public safety has always been an area of great importance for every country, and, in recent years, various types of water shooting robots have been developed towards intelligence and automation [7]. One way for the intelligent development of the water shooting robot is to use the control computer to collect information from various sensors, and adjust various parameters of the water jet based on this information to achieve the goal of intelligent and automated shooting. Table 1 shows the sensor and highlight functions of several current intelligent systems [8,9,10,11] and the intelligent water shooting robot involved in this paper.

**Figure 1:**
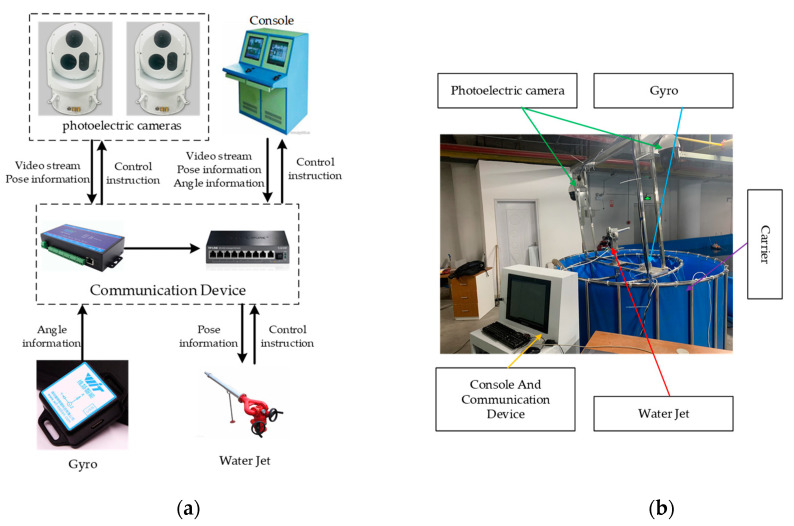
System hardware and physical platform. (**a**) Hardware module diagram; (**b**) Picture of the physical platform.

The intelligent water shooting robot involved in this paper is designed as a general shooting system, which can be applied to firefighting, maritime affairs, anti-terrorism, and other fields. The system can track and lock on to the target selected by the operator and shoot it automatically. Its basic hardware composition is shown in Figure 1a. Two photoelectric cameras and a gyroscope are used as the sensors of the system to collect various types of environment information. Dual photoelectric cameras are used for distance measurement; high-precision gyroscopes are used to solve the problem of muzzle shaking when the carrier shakes, we adopt the method in [12,13] to read the gyroscope’s data through the serial port server and Kalman filtering is used to remove noises. Then, the pose of the water jet is compensated by the pose and motion acceleration of the carrier obtained from the processed data, to keep the muzzle stable. The console provides the operating environment for the system software, collects and processes sensor information, and controls the water jet, and the communication equipment is responsible for data transfer between various hardware devices. In this experimental device, we use two blue buckets of different sizes to simulate the scene of carrier shake: the intelligent water shooting robot is fixedly installed on the small bucket and placed inside the large bucket, and 2/3 of its water volume is injected into the large bucket. At this time, the small bucket is floating on the water surface of the big bucket. When an external force is applied to the intelligent water shooting robot, the small bucket can be shaken to simulate the movement of the intelligent water shooting robot carrier. The picture of the physical platform is shown in Figure 1b.

**Figure 2:**
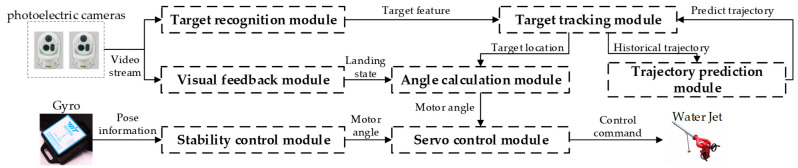
System software module.

The software module interaction of the system is shown in Figure 2. The system software modules include seven modules: target recognition module, target tracking module, trajectory prediction module, angle calculation module, stability control module, visual feedback module, and servo control module. The operation procedure can be summarized as following 6 steps:

Step 1: Target recognition. Detects and recognizes the target selected by the operator on the UI (user interface) through the video frame acquired by the photoelectric camera, and then generates the target feature.

Step 2: Target tracking. Finds the position of the target on the video frame according to the target features, then adjusts the pose of the photoelectric camera to keep the target in the center of the video frame. After that, the position (in terms of coordinate) of the target in water jet coordinates is computed according to the pose of the photoelectric camera and the historical trajectory (contiguous historical positions mentioned above) of the target is recorded.

Step 3: Trajectory prediction. The feature trajectory is predicted according to the historical trajectory of the target, and then the pose of the photoelectric camera is adjusted to pre-lock it according to the feature trajectory.

Step 4: Stable control. The compensation angle of each joint motor according to the pose of the carrier acquired by the gyroscope is computed, and then the angle is sent to the servo system to control the water jet to keep the muzzle stable in word coordinates.

Step 5: Angle calculation. The yaw angle is calculated using a geometric method and the pitch angle using a pitching angle model according to the position of the target, and they are sent to the servo system to adjust the water jet. The fluid is then launched to shoot the desired target. The model’s parameters are updated through the back-propagation according to the state relationship if the state relationship of the visual feedback module is received.

Step 6: Visual feedback. Jet trajectory segmentation is performed on the video frame captured by the photoelectric camera, and the jet landing point is calculated according to the segmentation result. The state relationship between the landing point and the target is determined according to the position of the landing point and target, and then the result is fed back into the angle calculation module, after which the operation returns to Step 2.

The intelligent water shooting robot needs to be able to automatically adjust the yaw angle and pitch angle of the water jet to shoot the desired target according to the specific location of the desired target. In order to achieve that, it is important to get an accurate trajectory model, and then use the trajectory model to adjust the water jet parameters for shooting [14,15,16]. The trajectory model is non-linear, which is affected by many external factors, such as changes in wind speed or pump power, jet carrier shake [17,18], etc. Meanwhile, the water flow will become scattered after a short distance of movement, which increases the difficulty of modeling.

The main contribution of this paper is to propose a novel method to establish and update the jet trajectory model according to the actual environment, so as to accurately calculate the pitch angle of the water jet according to the specific location of the desired target. The parameters of the pitch angle model are updated in real-time according to the landing point state information feedback by the visual feedback module, so that the jet model can dynamically adapt to the influence of the environment.

The chapters of this paper are organized as follows: Section 2 briefly reviews related works concerning the methods of constructing the trajectory model. Section 3 introduces the model in detail, including the offline fitting model and the detailed process of the online updating model. Section 4 introduces the process of the experiment, gives the detailed experimental results, and analyzes and compares the experimental results. Finally, some conclusions are given in Section 5.

## 2. Related Works

At present, methods to construct the trajectory model can be divided into two types. One is based on mathematical modeling and the other is based on deep learning.

The method based on mathematical modeling is generally based on Newton’s second law. Those methods were based on the assumption that the motion of water jet trajectory can be decomposed to the sub-component from the horizontal direction and vertical direction, and the horizontal and vertical direction movement do not affect each other. Zhang et al. [19] and Min et al. [20] consider the influence of air resistance and gravity to mathematically model the jet trajectory based on Newton’s second law. This method is not universal, but it can achieve high accuracy for specific devices. However, the accuracy rate cannot be high because of the limited number of factors considered. In addition, due to the scatter of the water jet, the water jet is essentially different from the flight of solids such as projectiles, and the particle trajectory method cannot be used to describe it [4]. In order to solve this problem, Xiang et al. [5] modeled the flight trajectory of a single water droplet. They adopted an exponential growth model of air resistance and proposed a jet trajectory prediction method with only one undetermined coefficient *b*. They calculated the jet trajectories for several *b* values under various conditions. This method is partly universal, but it lacks robustness for the environment and does not consider the mutual influence between water droplets. Zhu et al. [6], based on the moving particle semi-implicit (MPS) method, considered the initial velocity of the droplet and the relationship between the velocity and the air resistance, and modeled the flight trajectory of a single droplet. The MPS is a mesh-free method for calculating incompressible fluids [21], and is widely used for complex fluid modeling and can be used for coupling analysis of fluid or rigid body and powder [22,23,24]. This method considers the mutual influence between water droplets, and uses a kernel function to calculate the interaction force between water droplets. It can achieve higher prediction accuracy when the initial jet velocity is low. However, the increase in the air resistance of the water droplets will greatly affect the accuracy of the jet modeling.

Methods based on deep learning generally use neural networks to fit the model, which reduces the amount of manual calculation and is simple to implement. Zhang et al. [25] and Li et al. [26] used the back-propagation neural network model to predict the landing point of the water jet, taking into account the muzzle height, jet pressure, wind direction, and other characteristics of the water jet and used them as the input of the model, the model outputs the coordinates of the jet’s landing point. They designed a three-layer network model and changed the number of nodes in the hidden layer many times to find the optimal solution for the model’s calculation speed and accuracy. Zhang et al. [25] also used genetic algorithms to optimize the network model to solve the problems of slow model convergence and local optimal solutions. Deep learning methods are highly accurate in specific environments, but cannot respond fast enough to changing external environments. At the same time, since deep learning is data-driven, the trained model relies too much on the dataset. The prediction effect is good when the actual environment is similar to the training dataset, but if the environment is unknown, the model’s poor generalization ability will cause a great decrease in shooting accuracy.

Current methods have a common problem as the trajectory model does not have the ability to fine-tune online according to the actual environment. Due to the change of wind speed, change of pump power, and jet carrier movement, the actual distance between the water jet muzzle and the expected landing point will change. In addition, due to the backhaul error caused by the clearance between the meshing gears in the process of mechanical transmission, these errors will also affect the control accuracy of the water shooting robot [27,28,29,30]. Once the model established for the ideal environment is affected by these factors, there will be a gap between the predicted pitch angle and the required pitch angle, which means that the water jet cannot shoot the target accurately according to the predicted pitch angle.

Inspired by the traditional fitting algorithm [31] and the back-propagation algorithm [32] in deep learning, in this paper, an online tuning model for the water jet landing point based on the back-propagation algorithm is proposed to solve the above problems. The proposed model’s parameters can be adjusted dynamically according to the actual application environment to dynamically eliminate external disturbances and achieve accurate shooting. The proposed method includes two stages of offline preparation and online adjustment. On the offline preparation stage, a series of discrete data of water jet pitch angle and its corresponding distance were collected manually. Then, a preliminary curve function is generated by those offline data. On the online adjustment stage, firstly, the water jet begins to shoot the desired target using the curve function which was generated by stage one, then, the location of the water flow landing point is detected by a vision system and the location bias between water flow landing point and the desired target is calculated. After that, the parameters of the curve function are adjusted using back-propagation algorithm and, finally, a fitting curve that fits perfectly in the current environment is generated. With the proposed model, there is no need for the user to consider the external factors of the current environment; it is simple and totally adaptive to the environment.

## 3. Methodology

An intelligent water shooting robot is a robot composed of two joints. The essence of precise control is to propose a kinematics inverse algorithm that is adaptive to changes in the external environment. The system includes a visual feedback module, which can obtain the status relationship of the landing point and the desired target in real-time. The core of the method in this section is to obtain the precise rotation angle of the two joints of the robot according to the landing point state of the visual feedback, so as to achieve precise shooting at the desired target. Therefore, this section will introduce the calculation process of the robot yaw angle and pitch angle required for precise control of the water jet, that is, the data that the angle calculation module of the system needs to calculate. The yaw angle is a precise theoretical result obtained by the geometric method based on the positional relationship between the water jet and the desired target. The pitch angle is the data predicted by the two-stage model we proposed.

### 3.1. Yaw Angle Calculation

**Figure 3:**
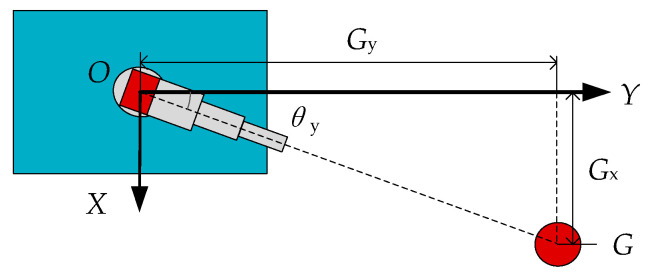
The coordinate relationship between the water jet and the desired target *G*.

In the water jet coordinate system, by analyzing the coordinate relationship between the water jet and the desired target *G*, the yaw angle is calculated using the geometric analysis method. The top view of the coordinate relationship between the water jet and the desired target *G* is shown in Figure 3.

When the water jet locks the target and prepares to shoot, the origin *O* of the coordinate system, the muzzle, and the center of the target are collinear. At this time, the angle between the barrel and the *Y*-axis is the yaw angle *θ*_y_. According to the geometric relationship shown in Figure 3, we can know the following:

(1) $${cos\theta }_{y}\;=\;\frac{G_{y}}{\sqrt{G_{x^{2}}+G_{y^{2}}}}$$

(2) $${sin\theta }_{y}\;=\;\frac{G_{x}}{\sqrt{G_{x^{2}}+G_{y^{2}}}}$$

Since the range of the yaw angle of this device is −90° to 90°, *cosθ*_y_ cannot be judged as positive or negative within this range, which will affect the subsequent solution of *θ*_y_. Therefore, we use Equation (2) to compute the yaw angle:

(3) $$\theta_{y}\;=\;\arcsin\frac{G_{x}}{\sqrt{G_{x^{2}}+G_{y^{2}}}}$$

### 3.2. Pitch Angle Calculation

The online tuning model of the water jet landing point based on the back-propagation algorithm includes two stages: using Matlab to fit the nonlinear function equation model offline and the online parameter adjustment method based on the back-propagation algorithm.

Stage I: Generate the initial prediction model: using Matlab to fit the nonlinear function equation offline model. We first collect a set of data of the pitch angle of the water jet under an ideal no-wind environment, constant pump power, and stationary carrier, and the distance between the corresponding jet landing point and the muzzle as an offline fitting dataset. Then, the polyfit function of MATLAB is used to fit a nonlinear mapping equation, that is, the initial prediction model, which is used in the initial calculation of water jet pitch angle.

Stage II: Update prediction model: using the online parameter adjustment method based on the back-propagation algorithm to update the prediction model in real-time. After the first shooting of the water jet using the angle result that is generated by Stage I, the intelligent system will receive the visual feedback of the state relationship between the landing point and the desired target, then a back-propagation algorithm is proposed to adjust the parameters of the prediction model in real-time according to the feedback state relationship.

#### 3.2.1. Generate Initial Prediction Model

We placed the water jet in an indoor environment with no shaking and no wind, keeping the pump power constant and the carrier stationary, and collecting the distance from the landing point to the muzzle corresponding to the pitch angle of the water jet within the range of −9° to 32°. This data range, covering the water jet’s shortest range to the farthest range, the acquisition resolution is 1°, each angle is collected 20 times, and the average distance is used as the final fitting data. The detailed data collected is shown in Table 2. Where *dis* is the distance from the landing point to the muzzle, and *deg* is the pitch angle of the water jet.

**Figure 4:**
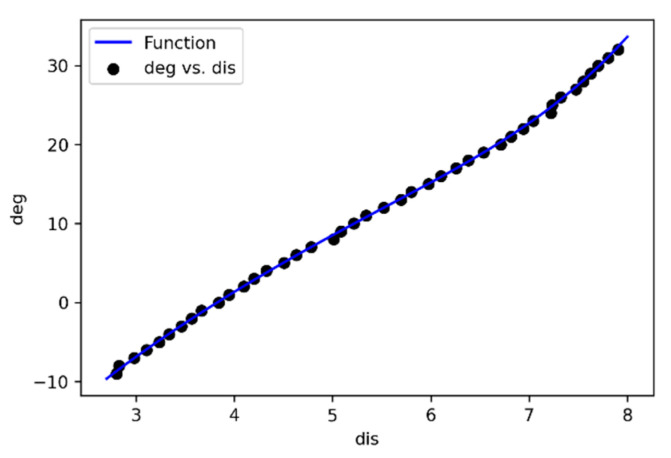
Matlab fitting result.

The polyfit function in Matlab has three parameters, the first parameter is *dis*, the second is *deg*, and the third is the order *n* of the polynomial that you want to fit. In the actual fitting test, we found that the 6th order equation has a better fitting effect on the jet data, so we set *n* to 6. The fitting result is shown in Figure 4.

The fitted nonlinear equation model is Equation (4), where *a*, *b*, *c*, *d*, *e*, *f*, *g* are the parameters fitted by the equation, there are 7 parameters in total, and the input *x* of the equation is the straight-line distance *dis* from the landing point to the coordinate origin of the water jet, the output of the equation *F*(*x*) is the pitch angle *deg* of the water jet.

*F*(*x*) *= ax*^6^ + *bx*^5^ + *cx*^4^ + *dx*^3^ + *ex*^2^ + *fx* + *g* (4)

#### 3.2.2. Update Prediction Model

**Figure 5:**
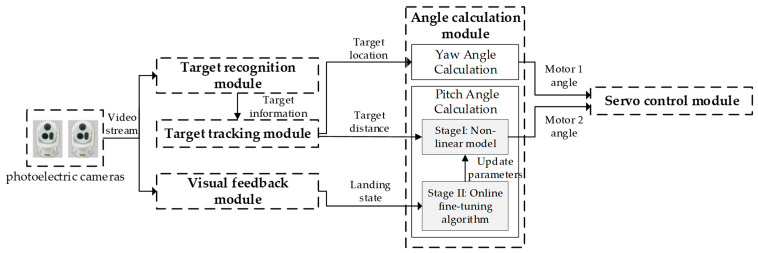
Process of online parameter adjustment method based on the back-propagation algorithm.

The process of the online parameter adjustment method based on the back-propagation algorithm is shown in Figure 5. The specific steps are as follows:

Step 1: The target tracking module calculates the target location according to the target information given by the target recognition module, and outputs the calculated distance between the target and the origin of the water jet coordinate system to the nonlinear equation model of the angle calculation module.

Step 2: The nonlinear equation model calculates the theoretical pitch angle that the water jet needs to adjust according to the input distance, and outputs the theoretical pitch angle to the servo control module.

Step 3: The servo control module adjusts the motor angle of the water jet according to the received pitch angle and executes the shooting command.

Step 4: The visual feedback module analyzes the shooting result (it includes 3 statuses: the landing point is located in front of the target, the landing point is behind the target, and the landing point hits the target) according to the video stream captured by the photoelectric camera, and feeds the shooting result back to the online fine-tuning algorithm.

Step 5: The online fine-tuning algorithm calculates and updates the parameters of the nonlinear equation model according to the shooting results feedback by the visual feedback module.

The online fine-tuning algorithm is based on the back-propagation algorithm, which calculates and updates the parameters of the nonlinear equation model fitted in Section 3.2.1 based on the feedback results in real-time. The steps of the back-propagation algorithm include constructing the loss function, calculating the derivative of the loss function and the model parameters, and updating the model parameters using the gradient descent method. The steps to fine-tune the algorithm online are as follows:

Construct a loss function. Use *S* to denote the target distance. Substituting *S* into Equation (4), the theoretical pitch angle adjustment angle *θ*_s_ can be obtained as follows:

*θ*_s_ = *F*(*S*) (5)

According to the shooting result feedback from the vision-based closed-loop control module, we construct a loss angle, denoted by *θ*, then the actual required pitch angle adjustment angle *θ*_gt_ is as follows:

*θ*_gt_ = *θ*_s_ + *θ* (6)

We use the mean square error (MSE) loss as the loss function of the back-propagation. The loss function is expressed as Equation (7), where *F*(*x*) is the nonlinear equation model of offline fitting, that is Equation (4):

*Loss* = (*θ*_gt_ − *F*(*x*))^2^ (7)

Calculate the derivative of the loss function and model parameters. Taking the derivative of the *Loss* with respect to *F*(*x*), Equation (8) is obtained:

(8) $$\frac{dLoss}{dF(x)}=\;-2\;\times \;(\theta_{\mathrm{gt}}\;-\;F(x))$$

Use vector *W* to represent the parameters in Equation (4):

*W* = [ *abcdefg* ]^−1^ (9)

According to the derivation rule of the matrix, the *F*(*x*) takes the derivative of the vector *W* to obtain the Equation (10):

(10) $$\frac{dF(x)}{dW}=\;{\left[\;x^{6}\;x^{5}\;x^{4}\;x^{3}\;x^{2}\;x\;1\right]}^{-1}$$

Find the derivative of the *Loss* with respect to the 7 parameters of *F*(*x*), that is, find the derivative of the *Loss* with respect to the vector *W*. According to the chain rule of the derivative, the simultaneous Equations (9) and (10) can be obtained by Equation (11):

(11) $$\frac{dLoss}{dW}\;=\;\frac{dLoss}{dF(x)}\;\times \;\frac{dF(x)}{dW}$$

Use gradient descent method to update model parameters. According to the gradient descent theory, the parameters of the model can be updated online according to Equation (12), where *lr* is the learning rate:

(12) $$W\;=\;W\;-\;lr\;\times \;\frac{dLoss}{dW}$$

## 4. Experiment

In the experimental part, we conducted simulation verification and the actual platform test. Simulation verification is to verify the correctness of the proposed method in the paper, and to check whether the online fine-tuning algorithm can adjust the parameters of the model correctly and in real-time according to the loss. In addition, we also compared the performance of the model with the neural network method in [25]; the test on the real platform is to verify the actual effect of the proposed two-stage model.

### 4.1. Simulation Verification

In the simulation verification, we abstracted the influence of external factors on the landing point into the change of the actual pitch angle required by the water jet, and carried out several groups of simulation experiments. We selected 50 test points evenly in the range of 2.9~7.3 m, and added a random angle between −2°~2° to the predicted pitch angle of the initial model as the actual pitch angle needed by the water jet to simulate three situations: the landing point is in front of the target, the landing point is behind the target, and it shoots the target.

**Figure 6:**
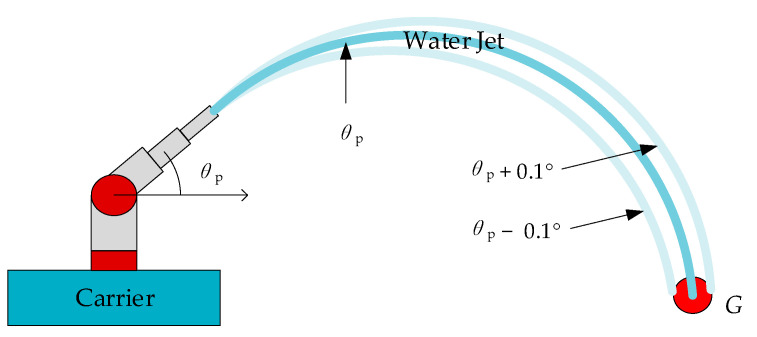
Schematic diagram of jet shooting.

In order to figure out the stopping conditions of the online iteration of the model, we conducted a pitch angle adjustment test on the actual machine platform, and found that when the pitch angle error of the water jet is within 0.1°, no matter the length of the distance between the desired target and the origin of the water jet coordinate system, the jet can accurately shoot the target, as shown in Figure 6, *G* is the desired target. We set the loss angle *θ* in Section 3.2.2 to 0.1°, and the iteration stop condition is set as the error angle between the model predicted pitch angle and the actual required pitch angle is less than 0.1°. Since the gradient of each iteration is related to the input value of the model, the larger the input value, the greater the gradient of each iteration, which leads to an excessively large update angle in each iteration and severe muzzle jitter. Therefore, we use the dynamic initial learning rate, the calculation formula of the initial learning rate is as follows:

*lr* = 0.1364 × *x*^−11.67^ (13)

**Figure 7:**
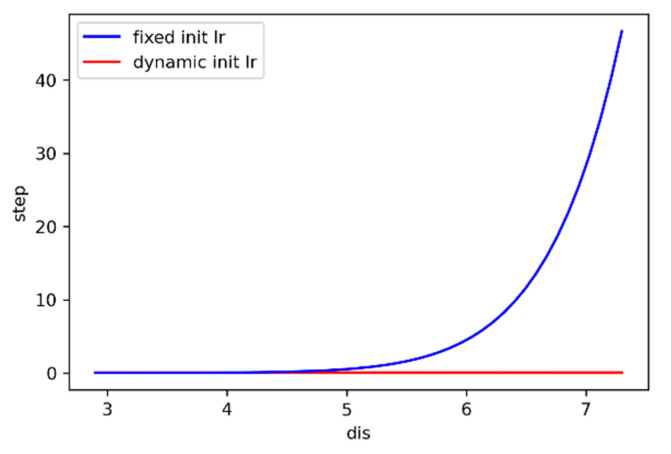
Relationship curve between the model input value and iteration step length.

The input *x* of the equation is the straight-line distance *dis* from the landing point to the muzzle, so that the initial angle iteration step is kept at 0.04°~0.06° to ensure that the muzzle will not shake sharply for every iteration. When the fixed initial learning rate and the dynamic initial learning rate are used, respectively, the influence curve of the input value *dis* on the step length is shown in Figure 7. It can be seen that the dynamic initial learning rate can make the model’s iterative step size have good robustness to the influence of the input value *dis*.

**Figure 8:**
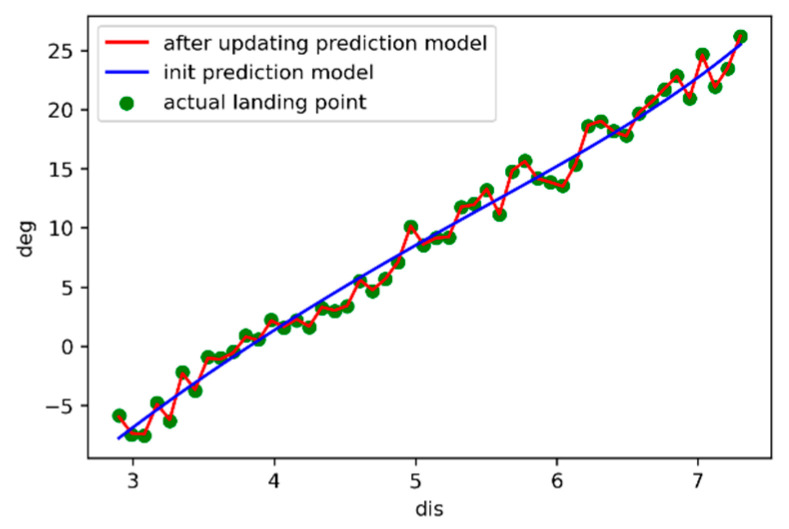
Curve of simulation experiment results.

In order to reduce the times of iterations required by the model while ensuring that the error can be reduced to within 0.1°, we dynamically adjusted the learning rate. The adjustment strategy is to double the learning rate if the model increases or decreases the output angle twice in a row during the iteration process. If the output angle is increased once in the two iterations and the output angle is decreased the other time, the learning rate is halved. The learning rate adjustment equation is as follows:

(14) lri={2×lri−1  if θi−1>θi−2>θi−312×lri−1 if θi−1>θi−2<θi−3 or θi−1<θi−2>θi−3 lri−1    otherwise

where *lr*_i_ indicates the learning rate of the *i*-th iteration, *θ_i_* indicates the *i*-th output angle. The results of the curve update with a random angle in the range of −2°~2° are shown in Figure 8. After the online update iteration, the initial model curve is slowly fitted from the blue curve to the actual green point, and gradually becomes a red curve. The results prove that this method can update the model parameters as accurately as the theoretical derivation.

In addition, we also changed the range of random angles to test the model’s performance in different error ranges. The specific results are shown in Table 3. The test was performed on the AMD-R5 3600 central processing unit (CPU). The iteration time represents the time required for the model output pitch angle error from the iteration start to the iteration to less than 0.1° at a certain test point, which means the calculation time required for the model to output the effective pitch angle each time. The error angle represents the difference between the predicted pitch angle output by the model after the iteration stops and the actual required pitch angle. Table 3 shows the average iteration time and average error angle of 50 test points under different random error conditions. The calculation formula is as follows:

(15) $$\mathrm{Mean}(P)\;=\;\frac{1}{N}\sum_{N}^{i}p_{i}\;\mathrm{where}\;p_{i}\;\in \;P$$

where *P* represents the set of test point iteration time or angle errors under each random error condition, and *N* represents the number of test points, which is 50. It can be seen from the table that the larger the range of increase of the random angle of error, the more iterations the model requires and the longer the iteration time will take. The error angle of each test point is between 0°~0.1°, and the average error angle is about 0.05°~0.06°.

Zhang et al. [25] used the GA-BP network to predict the jet’s landing point. They considered 7 factors such as muzzle height, horizontal angle, pitch angle, pressure, fluid rate, and wind. The GA-BP model has three layers: the input layer has 7 input nodes, and the input is the above 7 factors; the output layer has two output nodes, and the output is the *x* coordinate and *y* coordinate of the landing point; the hidden layer has 10 nodes. We compared various performance indicators with the neural network model used in [25], and the results are shown in Table 4. Since the output of the proposed model is the pitch angle, and the GA-BP model evaluation standard is the average distance between the theoretical landing point and the real landing point, we use the error angle of 0.1° to calculate the input distance on the iteratively completed model, to compute the distance error of the landing point, it is calculated as 0.11 m. When the average landing error distance is less than 0.34 m, the parameters of the GA-BP model are 14.5 times the proposed model parameters, the forward calculation time is 6.9 times and the calculation time for back-propagation is 200 times longer than the proposed model.

### 4.2. Test On Actual Platform

**Figure 9:**
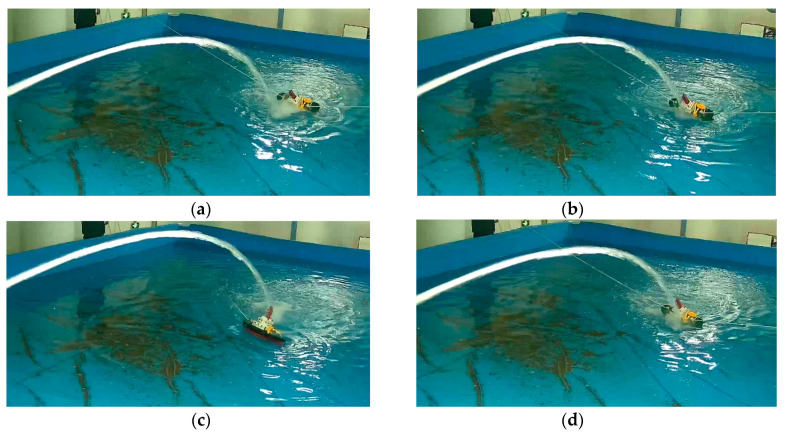
Actual platform test results. (**a**) The jet falls in front of the target; (**b**) Shoot the target after adjustment; (**c**) The jet falls behind the target; (**d**) Shoot the target after adjustment.

The screenshot of the test process of the actual platform is shown in Figure 9. (a) and (b) show that when the jet falls in front of the target, the pitch angle of the water jet is adjusted by this method and then accurately shoots the target; (c) and (d) show that the jet falls behind the target, the pitch angle of the water jet is adjusted by this method and then accurately shoots the target.

We have counted the number of model iterations and the time required for iteration when there is a measurement error of 0.5 m at different distances. As shown in Table 5, the actual distance represents the actual distance between the desired target and the origin of the water jet coordinate system, and the measured distance represents the measured distance between the desired target and the origin of the water jet coordinate system measured by the software algorithm, we add an error of ±0.5 m to the measured distance to simulate the influence of various factors on the jet. The time required for each iteration is the time of landing point detection. On the GTX TITAN X GPU (graphic processing unit), the average landing point detection time for processing one frame is 37 milliseconds. The model forward calculation and backward calculation time are far less than the landing point detection time, so we ignore them. Table 5 shows the experimental results of the fixed initial learning rate and the dynamic initial learning rate. It can be seen that the dynamic initial learning rate enhances the robustness of the model iteration process to the input distance under the condition that the time required for the iteration is almost the same. In addition, the iteration required time within 300 milliseconds ensures a good effect of the practical application of the method and meets the real-time requirements of the intelligent water shooting robot.

## 5. Conclusions

An intelligent water shooting robot system that is equipped with a motion carrier and can accurately shoot a moving target is designed in this paper. In particular, in order to improve the accuracy of shooting the desired target, a two-stage parameter-adjusted water jet landing point prediction model is proposed. Firstly, a preliminary nonlinear equation model is fitted offline, and then the model is used online and the parameters of the preliminary equation model can be adaptively adjusted by the back-propagation algorithm according to the state relationship between the landing point and the target, which feedbacks from a visual system. The experimental results show that the proposed method can adjust the parameters effectively. Compared to the state-of-the-art model of GA-BP, the proposed model keeps the pitch angle error within 0.1° while with low model parameters, less forward propagation time cost, and less back-propagation calculation time. Applying the proposed method to the actual experimental device, when the shooting error distance reaches 0.5 m, the model can complete iteration within 0.3 s, and the jet can accurately shoot the desired target. The proposed method provides a new idea for computing the pitch angle of the water jet, and solves the problem that the mathematical modeling and neural network methods cannot dynamically update the model according to the real-time state information. In the future, we plan to conduct outdoor tests in real conditions under different outdoor weather conditions (cloudy, rainy, sunny, and weather with different winds and directions).

## Figures and Tables

**Table 1:** Sensor parameters and highlights of the intelligent system.

Model	Country	Sensors					Highlight Function
		Infrared	Camera	LIDAR	UAV	Gyro	
ScrumForce	Japan		√		√		Aerial and ground fused perspectives for fire detection
Colossus	France	√	√				Firefighting for narrow terrain
WalkMan2.0	Italy	√	√	√			Excellent fire environment awareness
THOR	America	√	√	√			Smoke-filled firefighting
Thermite3.0	America	√	√				Remote monitoring of dangerous fire scenes
RXRM50BD	China	√	√				Fire detection and tracking
Ours	China		√			√	Automatic tracking, locking, and trajectory prediction of the desired target in the situations of carrier shake and target movement

**Table 2:** The measured pitch angle and the corresponding landing point distance.

***deg* (°) **	**−9**	**−8**	**−7**	**−6**	**−5**	**−4**	**−3**	**−2**	**−1**	**0**	**1**	**2**	**3**	**4**
***dis* (m) **	2.80	2.83	2.98	3.11	3.23	3.33	3.46	3.56	3.67	3.84	3.94	4.10	4.20	4.33
***deg* (°) **	5	6	7	8	9	10	11	12	13	14	15	16	17	18
***dis* (m) **	4.50	4.63	4.78	5.01	5.09	5.21	5.34	5.52	5.70	5.80	5.98	6.10	6.26	6.38
***deg* (°) **	19	20	21	22	23	24	25	26	27	28	29	30	31	32
***dis* (m) **	6.54	6.71	6.82	6.94	7.04	7.22	7.23	7.32	7.48	7.55	7.63	7.70	7.81	7.91

**Table 3:** Simulation result of model performance.

	Angle	−1°~1°	−2°~2°	−3°~3°	−4°~4°	−5°~5°
Standard						
**Mean Iteration Time**		0.82 ms	2.12 ms	3.92 ms	5.30 ms	6.06 ms
**Mean Error Angle**		0.05°	0.06°	0.06°	0.06°	0.06°

**Table 4:** Comparison result with GA-BP model.

	Standard	Parameters	Forward Time	Backward Time	Average Error Distance
Model					
**GA-BP**		102	55 μs	1000 μs	0.34 m
**Ours**		7	8 μs	5 μs	0.11 m

**Table 5:** Number of iterations and required time at different distances.

Actual Distance (m)	Measured Distance (m)	Fixed Initial Learning Rate		Dynamic Initial Learning Rate	
		Number of Iterations	Required Time (ms)	Number of Iterations	Required Time (ms)
3.5	3	14	518	8	296
	4	9	333	8	296
4	3.5	11	407	8	296
	4.5	8	296	8	296
4.5	4	9	333	8	296
	5	5	185	6	222
5	4.5	8	296	8	296
	5.5	2	74	6	222
5.5	5	5	185	8	296
	6	1	37	8	296
6	5.5	3	111	8	296
	6.5	2	74	8	296
6.5	6	1	37	8	296
	7	4	148	7	259
7	6.5	2	74	7	259
	7.5	5	185	7	259

## Data Availability

Not applicable.
